# A case of Brunner’s gland adenoma mimicking tumors induced from head of the pancreas

**DOI:** 10.11604/pamj.2018.29.78.11502

**Published:** 2018-01-25

**Authors:** Hasan Bostanci, Kursat Dikmen, Ozgur Ekinci, Cagrι Buyukkasap, Mustafa Kerem

**Affiliations:** 1 Department of General Surgery, Gazi University Medical Faculty, Besevler, Ankara, Turkey; 2 Department of Pathology, Gazi University Medical Faculty, Besevler, Ankara, Turkey

**Keywords:** Brunner gland adenoma, mimicing, pancreatic head tumour

## Abstract

Brunner's Gland Adenoma is a very rarely seen benign tumor of duodenum. While it generally leads to obstruction and bleeding complaints, it may very rarely occur by mimicking a pancreatic tumor. A 48 years old male patient admitted to the gastroenterology clinic due to the epigastric pain spreading dorsally. No significant feature is present in his clinical history. A lesion containing cystic solid components in the size of 30x40 mm was detected in the head of pancreas as a result of the abdominal tomography. In the light of these findings, pancreaticoduodenectomy is applied to the patient. It is observed that tumor is in submucosal location and widely invaded the pancreatic head. In the histopathological examination, Brunner's Gland Adenoma is reported in pancreatic head localization. In this manuscript a case of Brunner's gland adenoma diagnosed by performing pancreaticoduodenectomy due to the mass in the head of the pancreas is presented.

## Introduction

The Brunner glands were first described by Johan Conrad Brunner, a Swedish anatomist, in 1688. The Brunner gland adenoma (BGA), on the other hand, was first described by Curveilheir in 1835 [1]. The BG adenomas are very rarely observed benign tumors in duodenum. They were reported by 0.008% in a series of 21,500 postmortem examinations [2]. The BGA often tend to develop on the posterior wall of the proximal duodenum, and are known as the submucosal structures of the duodenum. Even though their etiology is not exactly known, the hyperplasia of the ductal and stromal structures of the exocrine glands on the proximal duodenum mucosa is considered to be the underlying reason [3,4]. Most of those adenomas are small in size and progress asymptomatically. They can be seen incidentally during endoscopy. Most of the cases, in which those adenomas reach larger sizes and become visible, happen to be cases of hemorrhage or obstruction [5]. Other cases, where they reach such large sizes, may clinically mimic the tumors in the head of pancreas. In this case, we intend to present a BGA case that mimics a tumor in the head of pancreas.

## Patient and observation

**Figure 1:**
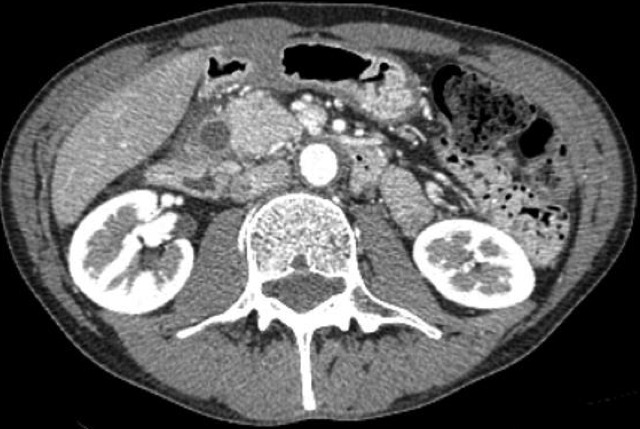
Abdominal CT image of the lesion in the head of pancreas

**Figure 2:**
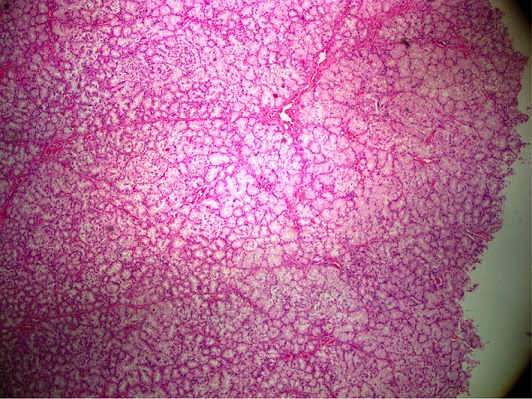
The low-power view of the tumor shows islands and sheets of crowded Brunner-gland-like acinar tumor cells, packed into larger nodules divided by seldom collagenous trabecules and thin-walled blood vessels. Magnification x 12,5; Hematoxylin & Eosin stain

**Figure 3:**
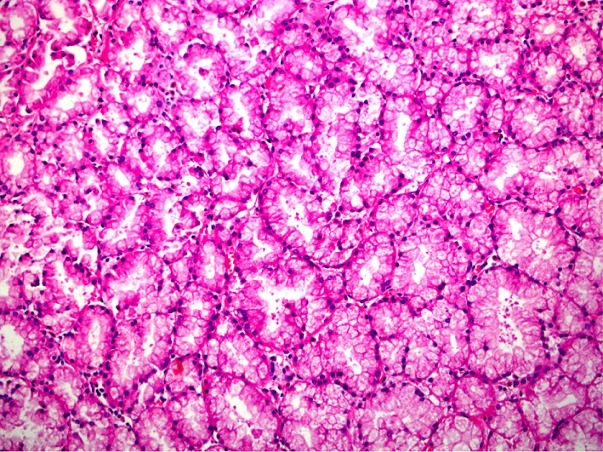
An higher-power view reveals very well differentiation of the tumoral cells towards the normal Brunner gland phenotype. There is no atypia or mitosis. The cells are monotonous, small, round and located at the bases of the cytoplasms. Magnification x 200; Hematoxylin & Eosin stain

The 48-year-old male patient applied to the gastroenterology clinic with the epigastric pain complaint. There were no characteristics in his background story. During the physical examination, sensitivity in the epigastric zone was detected. The routine blood test and tumor marker test results were normal. Any pathologies but antral gastritis were not detected as a result of the patient's upper gastrointestinal system endoscopy. A solid lesion which was in the size of 30 x 40 mm was detected in the head of pancreas and loss of fat plane between the pancreas and lesion, which raise doubt of a mass in the pancreas as a result of the abdominal tomography (Figure 1). There was expansion in the proximal wirsung canal of the mass. Surgical intervention was scheduled since the patient was symptomatic and on account of the solid content of the lesion. We performed pancreaticoduodenectomy, because we could not distinguished whether it was benign or malign tumor by CT findings. As a result of the macroscopic pathological examination of the specimen removed, a polypoid mass lesion with dimensions 4 x 3 cm that retained the 0.7 cm proximal in the duodenum wall of the oddi sphincter at medial and anterior was observed. It was observed that the lesion was located on the tumor's submucosa and expanded on the head of pancreas. The BGA presence was reported on the localization of the head of pancreas as a result of the histopatologic examination (Figure 2, Figure 3). Besides, no findings supporting malignity was observed and there were 14 reactive lymph nodes on this specimen. The patient was discharged on the 7th day following the operation.

## Discussion

The BGA develops from the Brunner glands located in the deep mucosa and the submucosa. Since the Brunner glands are widespread on the proximal duodenum, the BGA usually (in 57% to 70% of the cases) develops from the duodenum medulla [6]. In most of the cases, the BGA appears in the form of a BGA polypoid mass, usually stemmed (88%) and in the size of 1-2 cm. The gigantic BGAs in the size of several centimeters have been rarely reported [6]. On the other hand, the lesions smaller than 1 cm are referred to as the Brunner Gland Hyperplasia. The BGA are generally observed during the 5^th^ and the 6^th^ decades and at equal rates with both genders. The findings from the clinical examination of patients may include upper gastrointestinal hemorrhage, obstruction, vomiting, stomachache and diarrhea [1]. Since the condition is observed in most of the gastrointestinal hemorrhage cases, anemia can be spotted as the immediate symptom [6]. The condition may even lead to acute pancreatitis and, in cases localized near the ampulla of vater, to obstructive jaundice [7-9]. In our case, the patient suffered from non-specific abdomen ache rather than the said symptoms or findings. While the etiology and pathophysiology of the BGA are not yet known, it is considered that enhanced acid secretion causes the gland hyperplasia and induces the pathophysiological process [1]. Franzin et al suggested that, in patients with chronic gastric erosion and duodenal ulcer, there is a relation between the BGA and hyperchlorhydria [10]. However; Spellberg et al reported that the acid secretion inhibitors did not ensure regression in the lesion [11]. Another theory suggests that those lesions develop in connection with the intensive inflammatory cell infiltration, which theory was, on the other hand, proven wrong since lymphocytes are normally located on the submucosa of the intestinal tract. Besides, it was considered that Helicobacter Pylori could be playing a role in BGA pathogenesis [12]. It was reported as a result of a recent study conducted on 19,100 persons that Helicobacter Pylori genesis was detected to be positive in some 70% of the cases with BGA [12]. However, no Helicobacter Pylori pathogenesis was detected in our case.

BGA is a benign lesion with a good long term prognosis. It was reported within the literature that it could turn malign even to a rare extent [13]. The BGA generally tend to be small but could also attain large sizes. For diagnosis purposes, the symptom-oriented examination results, which are frequently observed especially by way of upper gastrointestinal endoscopy, are used. Even though computerized tomography and other radiologic imaging methods can be usefully employed for researching the etiology of existing symptoms; such methods are of non-specific nature. More importantly, those lesions could be confused with pancreoticoduodenal malignity during the imaging, which may lead to difficulties in diagnosis and cause the treatment strategy to be changed [14]. The matter of surgical excision of the BGA, which is still detected asymptomatically by coincidence at present, is controversial. While some authors believe that treatment is not required, some others argue that endoscopic or surgical resection is necessary to prevent the complications the BGA may cause. The BGA has been reported to cause acute hemorrhage and event shock in some cases [6]. Therefore, we recommend surgical or endoscopic resection for the treatment of the BGA. Endoscopic polypectomy should be the first choice if the tumor is small or stemmed. Open surgical excision is applied in the cases, for which endoscopic attempt fails or in which the tumor size is excessively large. The results of the operation are perfect, and no recurrence has been reported yet [15].

## Conclusion

We have presented a case of the BGA that mimicked a tumor located on the head of pancreas. The suspicion of malignancy is very high according to radiological findings. Even though it is very rare condition, its diagnosis and treatment should be planned carefully.

## Competing interests

The authors declare no competing interests.
